# Advancing IVF outcomes: AI-powered endometrial synchronizers to enhance implantation success

**DOI:** 10.1097/MS9.0000000000004338

**Published:** 2025-11-14

**Authors:** Muhammad Talha, Maliha Khalid, Laiba Shamim, Aminath Waafira

**Affiliations:** a Department of Medicine, King Edward Medical University, Lahore, Pakistan; b Department of Medicine, Jinnah Sindh Medical University, Karachi, Pakistan; c Department of Medicine, The Maldives National University, Malé, Maldives

*To the Editor*,

Implantation failure is still one of the most critical challenges for assisted reproductive technology (ART), especially IVF, in which the endometrial receptivity plays a major role in pregnancy success. Despite significant progress in culture and selection techniques for embryos, the rates of implantation mostly stagnate around 30–40%, mainly attributed to misalignment rather than nonembryo development and endometrial gating. This condition affects around 15% of patients globally undergoing IVF cycles^[1]^. This issue must be overcome for optimum improvement in IVF efficiency while minimizing emotional and financial burdens on patients.

Recent developments in artificial intelligence (AI) present solutions to improve endometrial receptivity evaluation. The AI-driven endometrial synchronizers employ machine learning algorithms to analyze ultrasound data and molecular data, providing accurate predictions of the implantation window. For instance, the Matris™ system processes thousands of imaging datasets to give objective, real-time recommendations for the optimal timing for embryo transfer, differentiating it from subjective ultrasound evaluations. Preliminary data from a prospective cohort study using Matris™ (*n* = 150, 95% CI [8%–22%]) demonstrate an improvement of up to 15% in implantation rate over traditional methods, though these findings require validation in larger, multicenter trials^[2]^. The applications of AI extend to personalized ovarian stimulation and embryo selection, thereby improving the overall efficiency of IVF by tailoring treatments to individual patient profiles and shortening time to pregnancy^[2,3]^. Limited evidence supports these applications, and challenges like algorithmic bias, cost, and access to low-resource settings need to be resolved to ensure equitable adoption. Furthermore, regulatory frameworks that include standardized validation protocols are key for ensuring safety and efficacy for clinical practice with AI tools^[2–4]^.

**HIGHLIGHTS**

- AI-powered endometrial synchronizers, such as Matris™, enhance implantation timing accuracy, improving IVF success rates by up to 15%.
- Machine learning models can outperform traditional methods in evaluating ovarian response, embryo viability, and personalized endometrial diagnostics.
- Adoption of AI in IVF requires standardized validation, clinician education, and collaborative policy development to ensure responsible clinical integration.

New studies indicate that AI models, like those powered by transcriptomic data, would likely outperform traditional prediction models in reproductive medicine, particularly in patients with recurring implantation failure. However, these results are still preliminary and need further validation for clinical application^[3–5]^. Support vector machines and neural networks have promising potential in predicting ovarian response and embryo viability, which could drive data-led decision-making in the clinic. Insights from other domains employing adaptive decision-making frameworks in the surgical fields strengthen the argument on AI’s ability to improve precision within ART. With data heterogeneity, algorithm transparency, and clinician training standing in the way, there are hurdles to overcome before AI can enter clinical practice^[5,6]^.

Although AI-driven endometrial synchronizers seem promising for potential improvement of IVF outcomes through precise individuated timings of embryo transfer, such gains should not be overstated on the current evidence base. The advent of AI into the field of assisted reproductive technology finds a strong path to refine endometrial assessments and improve outcomes through data-driven precision for IVF, even as challenges in implantation persist with technological advances. This argument, building on emerging studies, advocates for cautious optimism concerning AI synchronizers and similar tools, which, although having promise in small cohorts, need broader validation for their potential to counteract risks of bias and inequity in the discourse, bridging clinical potential with practical barriers unprecedentedly highlighted in reviews carried out earlier. Huge, multicenter trials designed to confirm the efficacy should now occupy the highest points, followed by the possible initiative of policymakers whose aim is to establish groups of partners feeding into standardized AI protocols, with ethically deployed systems. On their part, the clinicians need to engage in some directed training to facilitate the seamless integration of AI tools for easy access and safer innovations in infertility management^[7,8]^. Our work is in line with the TITAN Guidelines on the need for transparency in AI use in healthcare^[9]^.

## Data Availability

No data were generated for this manuscript.

## Research registration unique identifying number (UIN)

Name of the registry: N/A; Unique Identifying number or registration ID: N/A; Hyperlink to your specific registration (must be publicly accessible and will be checked): N/A.
